# Supplementary material for: Large-scale assembly of isotropic nanofiber aerogels based on columnar-equiaxed crystal transition
Source: Nat Commun. 2023 Sep 5;14:5410. doi: 10.1038/s41467-023-41087-y (PMC10480443; doi:10.1038/s41467-023-41087-y)
Supplement: Supplementary file 1 — Supplementary Information [file 41467_2023_41087_MOESM1_ESM.pdf]

Supplementary Materials for

Large-scale assembly of isotropic nanofiber aerogels based on columnar-equiaxed crystal transition

Lei Li, Yiqian Zhou, Yang Gao, Xuning Feng, Fangshu Zhang, Weiwei Li*, Bin Zhu, Shuo Tian, Peixun Fan, Minlin Zhong, Huichang Niu, Shanyu Zhao, Xiaoding Wei*, Jia Zhu*, Hui Wu*

*e-mail: huiwu@tsinghua.edu.cn (H.W.); jiazhu@nju.edu.cn (J.Z.); xdwei@pku.edu.cn (X.W.); liweiwei197@126.com (W.L.)

This PDF file includes:

Supplementary Text

Supplementary Figs. 1 to 22

Supplementary Tables 1 to 5

Supplementary Movies 1 to 11

Supplementary References (1 to 19)

Supplementary Text

1. Preparation process of alumina silicate nanofibers and different aerogels

Alumina silicate nanofibers

Alumina silicate nanofibers (ASNFs) were prepared by blow-spinning a PVA-templated Al−Si sol precursor[1]. First, 0.9 g PVA powder (Kuraray PVA-217) was dissolved in 5.1 g deionized water at 60 °C for 1 h. 3.49 g $AlCl_3 \cdot 6H_2O$ (Sinopharm), 3.12 g tetraethyl orthosilicate (TEOS, Macklin), and 8 g deionized water were mixed for 1 h to obtain clarified Al−Si sol via vigorous stirring. The Al−Si sol was subsequently added to the room-temperature PVA solution with stirring for another 1 h. After solution blow spinning (SBS), the obtained polymer-templated ceramic nanofibers were calcinated in a muffle furnace at 600 °C in the air at a 5 °C min^{-1} heating rate to remove residual organic components, yielding ASNFs with a characteristic diameter centered at ~200 nm and a composition of Al_2O_3:SiO_2 = 45wt%:55wt%.

PNF-based aerogels

Polyacrylonitrile (PAN) nanofibers were prepared by an industrial SBS method reported previously[1]. First, 6 g PAN powders (Mw = 250,000, DuPont) were dissolved into 44 g DMF (99.5%, Sinopharm) to obtain a uniform PAN/DMF solution. To perform the SBS process, the polymer solution was loaded into an integrated spinneret with 30 G needles and injected in a continuous stream at a speed of 1.5 mL h^{-1} (Supplementary Fig. 1). The airflow velocity was maintained constant at 10 m s^{-1} to provide an appropriate shearing force. The PAN nanofibers were collected in an air-permeable cage, positioned at a 30 cm distance from the spinneret. The obtained PAN nanofibers with an average diameter of 300 nm were dispersed with waterborne polyurethane (WPU) binders (35%, F0409, Jitian, China) in a deionized water/tertbutyl alcohol mixture (mass ratio of 5/1). The total dosage of PAN nanofibers and WPU were 0.5wt% and 0.35wt%,

respectively. A fiber disintegrator (50 rad s^{-1}, KLD-30, Aisirui, China) was used to prepare uniform nanofiber dispersions. Subsequently, the dispersion was rapidly transformed into nanofiber crushed ice through the crushed ice making machine. Finally, the obtained crushed ice was mixed with nanofiber dispersions in a mass ratio of 5:1 to fill the gap between crushed ice, transferred to the desired mold, frozen in a low-temperature chamber (–20 °C), and freeze-dried for 18 h to obtain the WPU-bonded PAN nanofiber aerogels.

p-ANF-based aerogels

To fabricate *p*-ANFs, 100 mL N-methyl pyrrolidone (NMP) was added to the reaction vessel and heated to 100 °C for 5 min under a nitrogen atmosphere[2]. Then, $CaCl_2$ powder was added and dissolved at 100 °C for another 30 min. After this, the reaction system was cooled to 15 °C, and dissolved *p*-phenylenediamine (PPD) with a stirring speed of 400 rpm. After cooling to 0 °C, terephthaloyl chloride (TPC) was further dissolved in the systems with a stirring speed of 2000 rpm. The reaction was stopped when the Weissenberg effect happened. Subsequently, the product was diluted by NMP, and added deionized water under high-speed homogenization (10 000 rpm). After being washed with deionized water several times, a *p*-ANF dispersion with a concentration of 0.1% was formed. Subsequently, the *p*-ANFs were dispersed in a deionized water/tertbutyl alcohol mixture with a mass ratio of 5/1. Finally, the dispersion with a solid content of 0.5% was rapidly transformed into nanofiber crushed ice and finally assembled as *p*-ANF aerogels using a similar process to PNF aerogels.

CNF- and CNT-based aerogels

The CNF dispersion (2wt%, Mujingling, China) was prepared by the TEMPO-oxidation method[3]. The CNF dispersion (0.5wt%) was directly used for ice crushing, re-casting and freeze-drying. The CNT dispersion (0.5 wt%, XF-Nano, China) and WPU binder (0.2wt%) were evenly mixed

and rapidly transformed into nanofiber crushed ice and finally assembled as CNT aerogels using a similar process to PNF aerogels.

MXene- and CDs-hybrid ASNF aerogels

The aqueous dispersion of MXene was manufactured by the etching and mechanical delamination process of the Ti_3AlC_2 MAX. Firstly, 2.0 g Ti_3AlC_2 MAX was added to 40 mL hydrochloric acid (HCl, 9 M, Sigma-Aldrich) dissolved with 3.2 g lithium fluoride (LiF, Sigma-Aldrich). After reacting at 35 ℃ for 24 h, the suspension was centrifuged at 3500 rpm and then formed a re-dispersed suspension with pH≈6. Then, the suspension was vigorously shaken for 30 min; and the supernatant MXene dispersion with a concentration of 0.1 wt% was obtained. MXene- and CDs-hybrid ASNF aerogels were constructed from ASNFs, CNFs, and MXene/CDs based on crushed ice casting and freeze-drying processes similar to ASNF aerogels.

2. Structural analysis of different aerogels

SEM analysis

Although similar preparation methods were applied, the micro-morphologies of the obtained porous materials were very different owing to the differences in surface properties and dimensions of the building blocks (Fig. 1d and Supplementary Fig. 3a). The PNF and *p*-ANF aerogels showed great heterogeneity, possibly owing to the hydrophobic property of the polymer surface. In contrast, a uniform cellular structure can be observed in CNF aerogels. The multi-laminated structure could be recorded in CNT aerogels with a single lamina of about 60 μm in thickness, composed of numerous CNT layers.

FT-IR analysis

The Fourier transform infrared (FT-IR) spectra of different aerogels were displayed in Supplementary Fig. 3b. Specifically, ASNF aerogels had two strong absorption peaks at 1200–850

cm^{-1} and 500–400 cm^{-1}, which were caused by the stretching vibration of Si-O bond. The weak and wide absorption band near 700 cm^{-1} was owing to the symmetrically stretching and vibrating of Si-O-Si. The splitting of the absorption peak was poor and the peak was smooth, because of Al element in ASNF aerogels entered the tetrahedron network and formed a tetrahedral skeleton like the Si element, resulting in the reduction of Si-O vibration frequency, the shift of the absorption peak to a lower wavenumber, and the widening of the absorption band. As for the *p*-ANF aerogels, the characteristic poly(p-phenylene terephthalamide) (PPTA) bands at 1647 (C-O stretching vibration), 1540 and 1251 cm^{-1} (N−H deformation and C−N stretching coupled mode) emerged in the spectrum of *p*-ANF. The FTIR spectrum of PNF aerogels showed $-CH_3-$ asymmetric stretching vibration at 2953 cm^{-1}, CH_2- asymmetric stretching vibration at 2917 cm^{-1}, CH_3 symmetric stretching vibration at 2873 cm^{-1}, CH_2 symmetric stretching vibration at 2845 cm^{-1}, CH_2 bending vibration at 1459 cm^{-1}, CH_3 symmetric deformation vibration at 1377 cm^{-1}, CH_3 out of plane rocking vibration at 1156 cm^{-1}, and CH_3 in-plane rocking vibration at 971 cm^{-1}. These peaks could correspond to the characteristics of the PAN molecular chain.

It is challenging to observe the characteristic absorption peak of CNT aerogels, but some characteristic absorption of WPU binders can be observed. In the spectrum, the C−O stretching vibration peak of amide I is at 1648 cm^{-1}, the N−H bending vibration peak of amide II is near 1541 cm^{-1}, the bending vibration peak of $-CH_3$ or $-CH_2$ is near 1402 cm^{-1}, and the C−N−C or C−O stretching vibration peak of amide V is near 1236 cm^{-1}. From the FTIR spectrum of CNF aerogels, the absorption peak of about 3500 cm^{-1} was the stretching vibration peak of alcohol hydroxyl. The absorption peaks around 1730 cm^{-1} referred to the stretching vibration of C=O, and three absorption peaks at 420, 1160, and 1110 cm^{-1} could denote the characteristics of type I cellulose.

<u>XPS analysis</u>

Evidence of the formation of different aerogels was obtained from the X-ray photoelectron spectroscopy (XPS) analysis (Supplementary Fig. 3c); the characteristic peaks of Al 2p (75 eV), Si 2p (105 eV), Si 2s (156 eV), and B 1s (180 eV) were assigned to Al- and B-doped silica tetrahedron structures.

TG analysis

To discover the thermal properties of different aerogels, TG was used to characterize the pyrolysis process at a heating rate of 5 K min^{-1} in an air atmosphere (Supplementary Fig. 3d). Specifically, the thermal decomposition of ASNF aerogels before 800 °C was negligible, owing to the superior thermal stability of ASNFs. As for the *p*-ANF aerogels, benefiting from the PPTA building blocks, they showed pronounced thermal stability compared to other polymers. The onset decomposition temperature of the *p*-ANF aerogels was 497 °C; the thermal decomposition was completed at 646 °C with an almost 100% weight loss. The thermogravimetric completion temperature of PNF aerogels was similar to that of *p*-ANF aerogels. However, its start point (~255 °C) was much earlier than that of *p*-ANF aerogels.

The slight weight loss from 50 to 200 °C in CNF aerogels could be owing to the evaporation of the physically absorbed water. A relatively large and rapid weight loss in the range of 200–317 °C was due to the carbonization of CNFs, while a slow weight loss was detected in the range of 317–500 °C due to the oxidation of the carbon skeleton. As for CNT aerogels, the weight loss location before 500 °C was caused primarily by the decomposition of WPU binders. The relatively large and rapid weight loss in the range of 500–564 °C was mainly generated from the oxidation of CNTs.

3. Electromagnetic shielding effect of MXene-hybrid ASNF aerogels

The ASNF aerogels served as potential skeletons for MXene, which effectively converted electromagnetic shielding function into macrostructures. As shown in the SEM images of Supplementary Fig. 4, the hierarchical structures of MXene-hybrid ASNF aerogels consisted of opened cells (size of 50–100 μm), semi-closed-cell walls (thickness of 0.5–2 μm), and entangled networks. The formation mechanism of the hierarchically entangled structures could be attributed to the phase transformation of the solvent and the co-assembly of ASNFs, CNFs, and MXene in the freeze-drying process. MXene-hybrid ASNF aerogels showed good microwave shielding performance. The EMI SE is a key index in EMI shielding related fields, and it can be expressed based on the *S*-parameters according to the following equations:

$$SE_T = SE_R + SE_A = 10\log\left(\frac{1}{|S_{21}|^2}\right) \quad \text{(S1)}$$

$$SE_R = 10\log\left(\frac{1}{1-|S_{11}|^2}\right) \quad \text{(S2)}$$

$$SE_{RA} = 10\log\left(\frac{1-|S_{11}|^2}{|S_{11}|^2}\right) \quad \text{(S3)}$$

As shown in Supplementary Fig. 4e, the highest EMI SE is 18.28 dB, corresponding to a specific EMI SE of ASNF aerogels reach ~3656 dB cm^3 g^{-1}, which is a relativity high EMI shielding parameter[4].

4. Fluorescence effect of CDs-hybrid ASNF aerogels

As shown in Supplementary Fig. 4f, PL peaks of CDs-hybrid ASNF aerogels were centered at 598 nm in the visible region, with a broad excitation range from 400 to 628 nm. This result confirmed that ASNF aerogels could serve as a potential skeleton for CDs, which effectively avoided fluorescence quenching and converted photoluminescence functions into the macrostructures

5. Temperature resistance analysis

The X-ray diffraction (XRD) results of the ASNF aerogels after calcination at 900, 1000, 1100, 1200, and 1300 °C were determined (Supplementary Fig. 14). Specifically, the amorphous peaks could be observed in the sample corresponding to a calcination temperature of 900 °C. For samples calcinated at temperatures of 1000 and 1100 °C, diffraction peaks in the XRD pattern were identified as the characteristic peaks of mullite with a one-to-one correspondence. After being calcinated at 1200 ℃, new diffraction peaks referred to cristobalite crystals could be observed at 21.9°. The sample from higher calcination temperatures had a more sharp and high-intensity crystallization peak, accompanied by the disappearance of an amorphous peak, confirming the crystallization of remaining amorphous components. After being long-term calcinated at 1200 ℃ for 24 h, a sharp peak of cristobalite crystals could be founded. However, with an average crystal size of 65.7 nm and crystallinity of 69% at this time, the nanograin-glassy dual-phase structure could also retain the high flexibility of ASNFs. When the calcination temperature exceeded 1300 ℃, the characteristic peak of cristobalite crystals significantly weakened with more mullite phase formation. We could observe many defects formed on the surface of ASNFs, along with the decrease of elasticity and the increase of density, the nanofibers would gradually become brittle and finally be crushed. The overlap of the diffraction peaks for crystal planes (120) and (210) clearly indicated the formation of mullite[5].

6. Preparation process of lamellar sponges and anisotropic aerogels

<u>Lamellar ASNF sponges</u>

ASNF sponges were prepared by SBS of a PVA-templated Al–Si sol precursor similar to the fabrication process of ASNFs as mentioned above. However, the concentration of sol will be increased appropriately. Specifically, 4.4 g $AlCl_3·6H_2O$, 4.0 g tetraethyl orthosilicate, and 8 g deionized water were mixed for 1 h to obtain clarified Al–Si sol *via* vigorous stirring. The obtained

16.4 g of Al−Si sol was subsequently added to 6 g of the room-temperature PVA solution (15 wt%) with stirring for another 1 h. The resultant lamellar ASNF sponges have a characteristic diameter centered at ~800 nm with a bulk density of 20 mg cm^{-3}.

Anisotropic ASNF aerogels

Typically, the used ASNFs nanofiber dispersion had identical composition to that for crushed ice manufacturing. The obtained dispersion was transferred to the desired mold, frozen on a cryogenic plate (–20 °C), and then freeze-dried for 18 h to obtain the anisotropic ASNF aerogel precursors. Next, the precursors were calcined at 600 °C for 30 min to prepare the anisotropic ASNF aerogels with a bulk density of 5.0 mg cm^{-3}.

7. Thermal conductivity calculation

In highly porous materials, there are three basic modes of heat transfer, which are convection (λ_{conv}), conduction [composed of λ_s (solid conduction) and λ_g (gaseous conduction)], and radiation (λ_{rad}) transfer. Convection is the macroscopic movement of the fluid, which leads to the mixture of the cold and hot parts to produce heat transfer. For highly porous media, when the pore diameter is lower than 4 mm, heat convection transfer could be neglected[6]. Conduction heat transfer is caused by the motion of microscopic energy carriers, such as molecules, atoms, free electrons, and phonons. Gaseous heat conduction is induced by the collision between gas molecules. The mean free path of air under the standard temperature and pressure is about 69 nm. It can be seen in Supplementary Table 3, no significant amount of mesopores was found in lamellar sponges. Their pore diameter could be much higher than the mean free path of the gas molecules. However, the mesopores in ASNF aerogels could effectively restrain the free movement of molecules and greatly decrease the gaseous heat conduction (Knudsen effect).

Solid conduction mainly depends on the lattice vibration of solid molecules around their equilibrium positions. In analogy with the concept of a photon in radiative heat transfer, the minimum quantization energy of lattice vibration is called a phonon. Take ASNFs as an example, its primary diameter is about 200 nm. Due to the size effect, the solid heat transfer and thermal conductivity of solid matrix could be significantly reduced. At the same time, the hierarchical mesoporous structures and tortuous cellular walls could also decrease solid heat transfer. The isotropic ASNF aerogels have an extremely low density and complex three-dimensional porous network structure. This kind of structure lengthens the path of heat transfer and increases the complexity of heat transfer through the solid matrix. Most materials emit radiant energy and transfer heat to a "cold" body after heating. Usually, the λ_{rad} of porous materials can be ignored at relatively low temperatures (＜800 °C).

The isotropic ASNF aerogels possess a high porosity and ultralow density with the characteristics of nanoscale internal pore and sub-microscale solid matrix. The effective thermal conductivity of lightweight materials mainly depends on the three-dimensional structure (shape, pore size, and spatial distribution) and intrinsic thermal properties of the materials. For typical ASNF materials, lowering λ_g and λ_{conv} are achieved by narrowing down the fiber diameter; and the decrease of λ_{sol} is usually achieved by decreasing the bulk density of the materials. Herein, we used the effective medium theory (EMT) equation to model the effective thermal conductivity (λ_E) on the premise that the material is isotropic[7, 8].

$$(1-\Phi)\frac{\lambda_s-\lambda_E}{\lambda_s+2\lambda_E}+\Phi\frac{\lambda_g-\lambda_E}{\lambda_g+2\lambda_E}=0 \quad \text{(S5)}$$

Where Φ is the porosity of different ASNF materials, λ_s is the thermal conductivity a bulk (no-porous) material with the same composition, which is about 2.1 W m^{-1} K^{-1}, λ_g is the gaseous thermal conductivity, which can be estimated according to:

$$\lambda_g = \frac{\lambda_{g0}\Phi}{1+2\beta K_n} \quad \text{(S6)}$$

where λ_{g0} is the gas conduction in free space (25 mW m^{-1} K^{-1}), β = 2, K_n is the Knudsen number[9].

$$K_n = \frac{l_m}{\delta} = \frac{1}{d\,(\frac{\sqrt{2}\pi P d_a^2}{\kappa_B T} + \frac{S\rho}{\Phi})} \quad \text{(S7)}$$

Which relates the mean free path l_m of a gas molecule to the pore diameter δ. Where d is the pore diameter, the isotropic ASNF aerogels include mesopores in the bonding point (3.86 nm), macropores in cell walls (800 nm), and foam pores (39.86 μm). P is the pressure, d_a is the diameter of the air molecule, S is the specific surface area, ρ is the density, κ_B is the Boltzmann constant and T is the temperature. The calculated effective thermal conductivity of isotropic aerogels was 19.53 mW m^{-1} K^{-1}, in good correspondence with the experimental values.

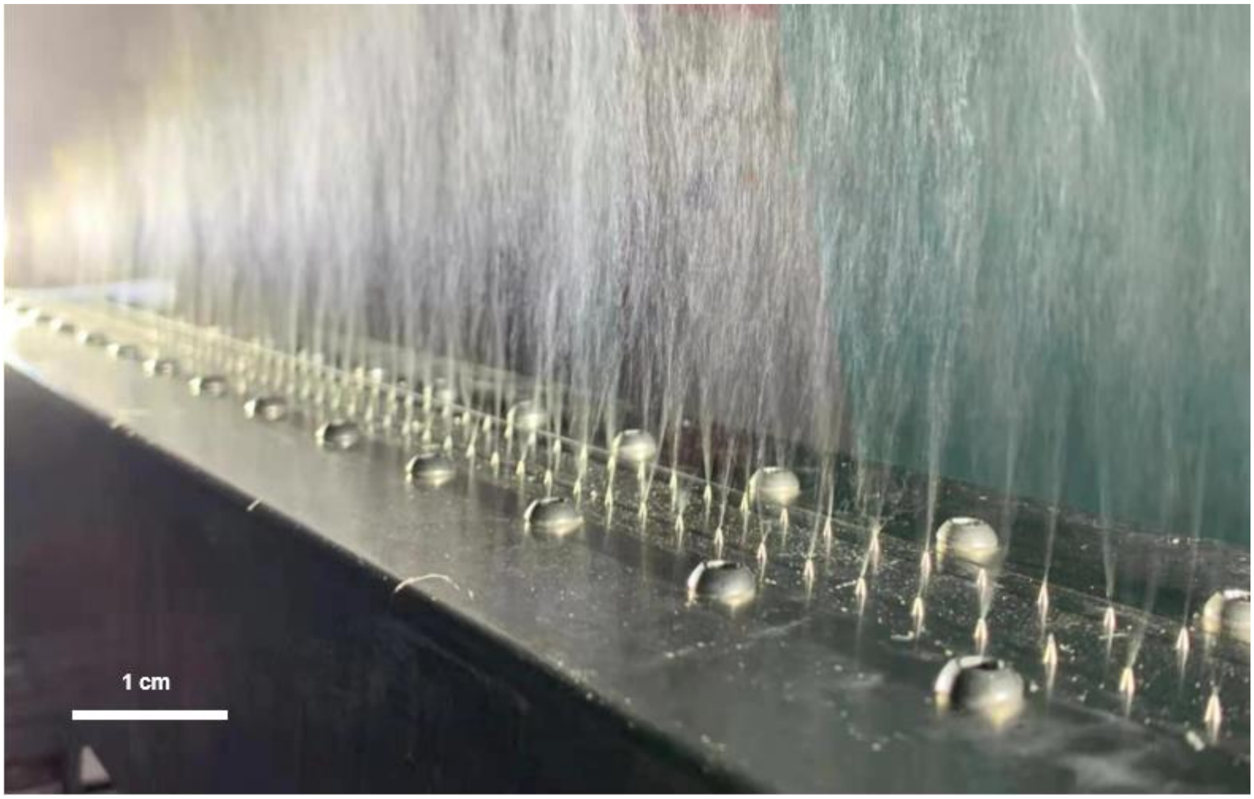

Supplementary Fig. 1. Multi-needle solution blow spinning equipment.

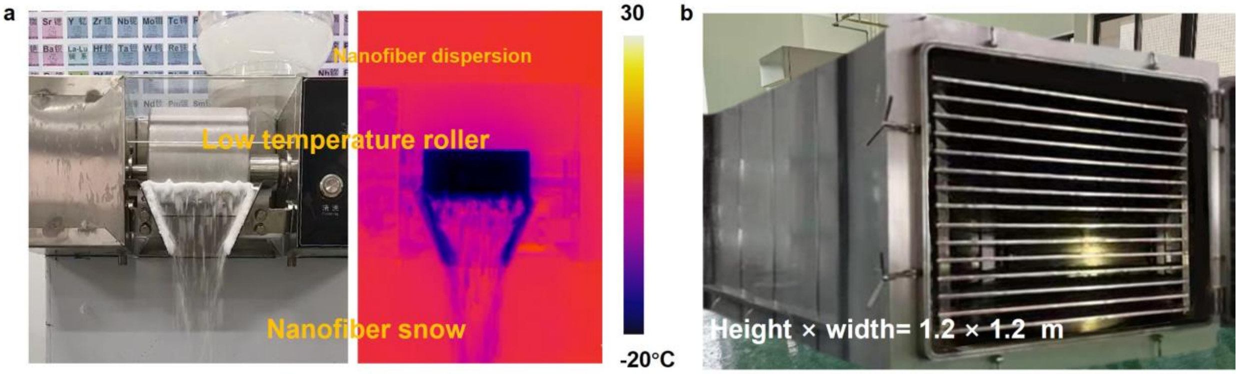

Supplementary Fig. 2. Manufacturing process of ASNF aerogels. a Nanofiber dispersion was rapidly frozen on the surface of the rotating cryogenic drum and scraped into crushed ice. **b** Photo of the industrialized freeze-drying equipment.

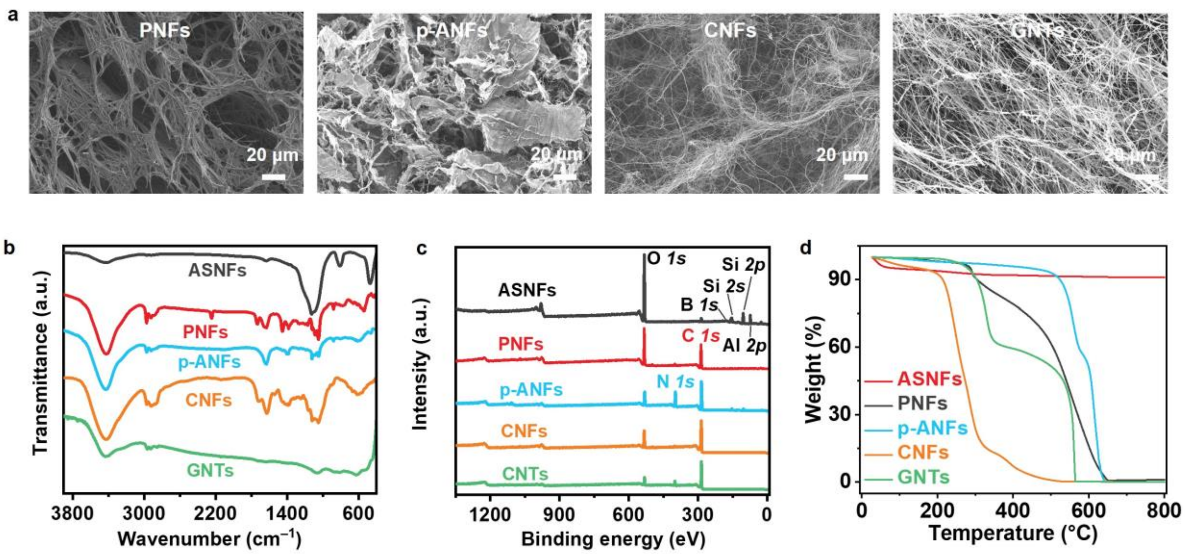

Supplementary Fig. 3. Generality of the crushed ice casting method. SEM images (**a**), FT-IR spectroscopy (**b**), XPS spectra (**c**), and TG results (**d**) of different aerogels assembled from different nanomaterials.

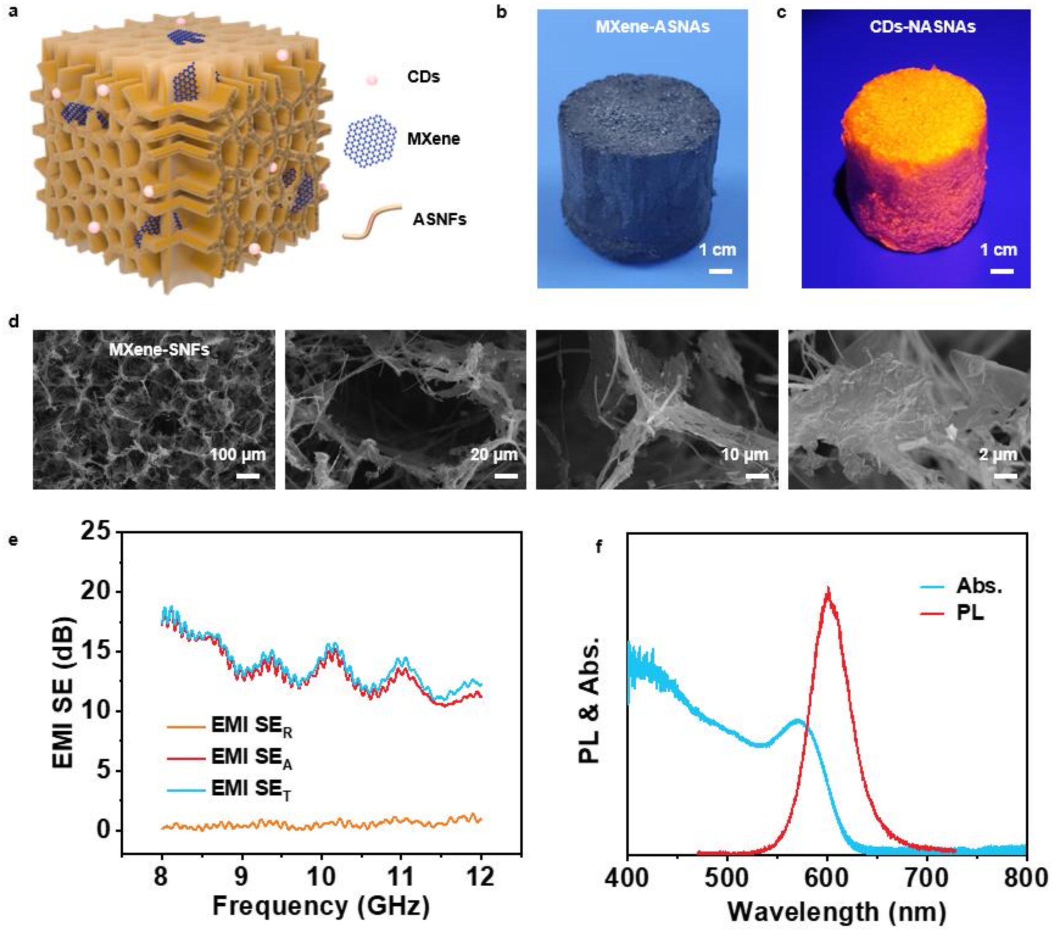

Supplementary Fig. 4. ASNF aerogels serve as a potential skeleton for different functional materials. a Schematic illustration of the structure of CDs- or MXene-hybrid ASNF aerogels. **b** Images of MXene-hybrid ASNF aerogels. **c** Optical images showing the photoluminescence function provided by CDs-hybrid ASNF aerogels. **d** SEM images of MXene-hybrid ASNF aerogels at different magnifications. **e** Electromagnetic shielding function provided by MXene-hybrid ASNF aerogels. **f** Photoluminescence function provided by CDs-hybrid ASNF aerogels.

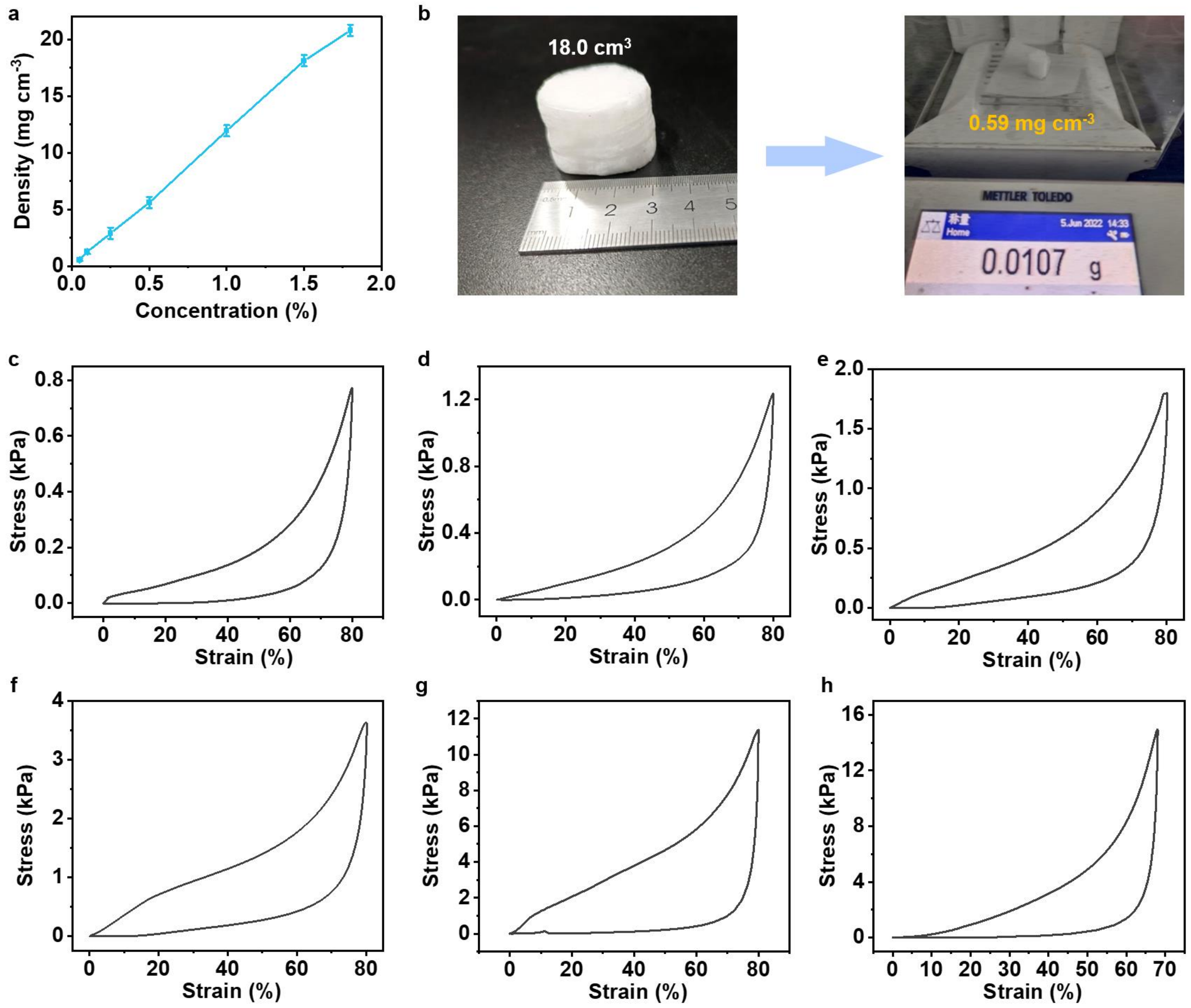

Supplementary Fig. 5. Density regulation of ASNF aerogels. a Density of aerogels *versus* concentration of nanofiber dispersion. **b** Weight measurement of ASNF aerogels. The sample with a volume of 18.0 cm^3 has a mass of 10.7 mg, which corresponds to a bulk density of 0.59 mg cm^{-3}. **c-h** Compressive stress-strain curves of ASNF aerogels with the densities of 1.0, 2.5, 5.0, 12.0, and 16.0 mg cm^{-3}.

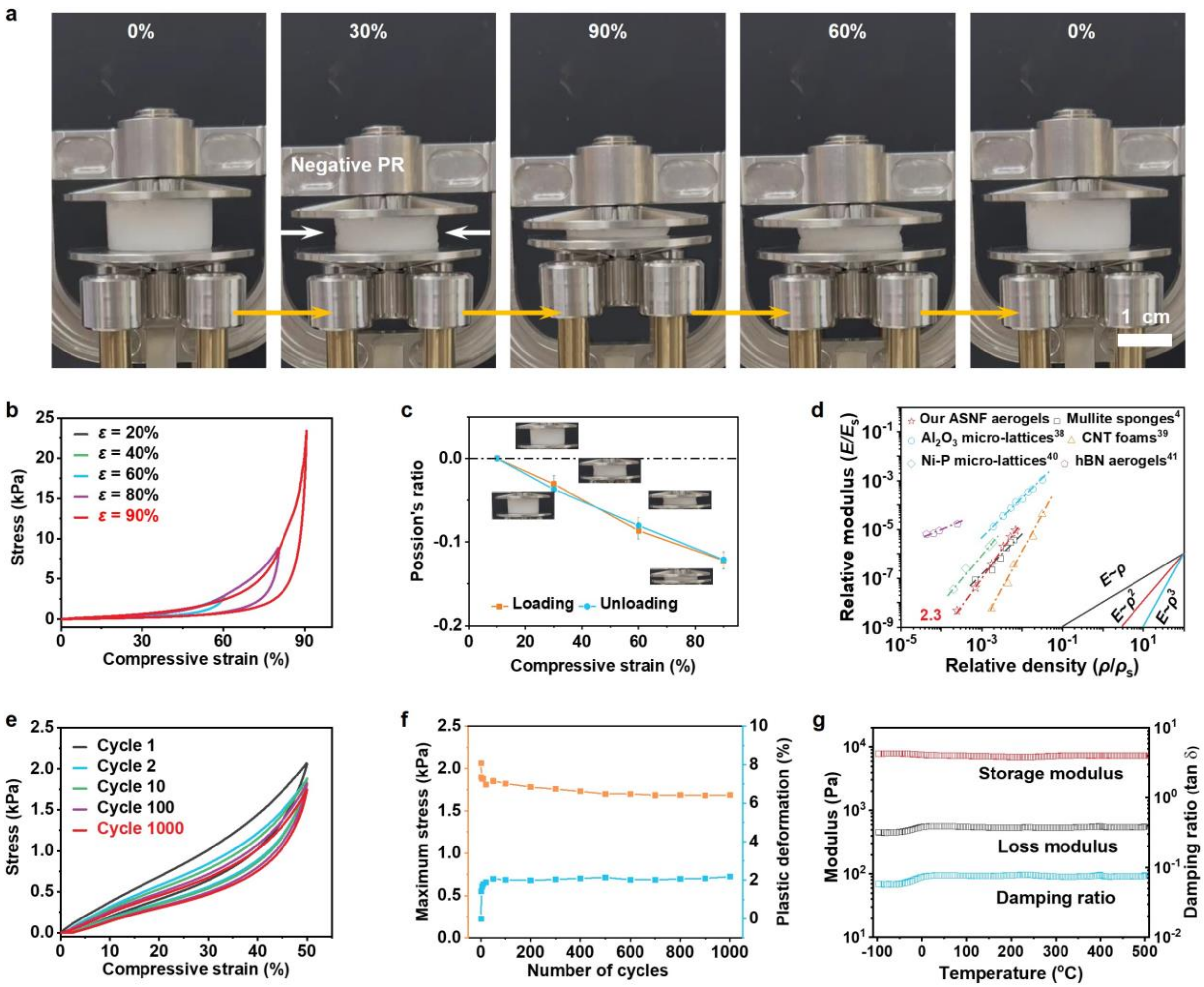

Supplementary Fig. 6. Mechanical performances. a Snapshots of one full cycle under 90%-compressive strain, demonstrating the negative Poisson's ratio (PR) of ASNF aerogels. **b** Compressive stress−strain curves during loading-unloading cycles with increasing strain amplitude. **c** Poisson's ratio of the ASNF aerogels versus strain. **d** Relative Young's modulus of selected materials with low densities. **e** A 1000-cycle test with a compressive strain of 50%. **f** Maximum stress and plastic deformation *versus* compressive cycles. **g** Temperature dependence of storage modulus, loss modulus, and damping ratio under an oscillatory strain of 3% at a fixed frequency of 1 Hz.

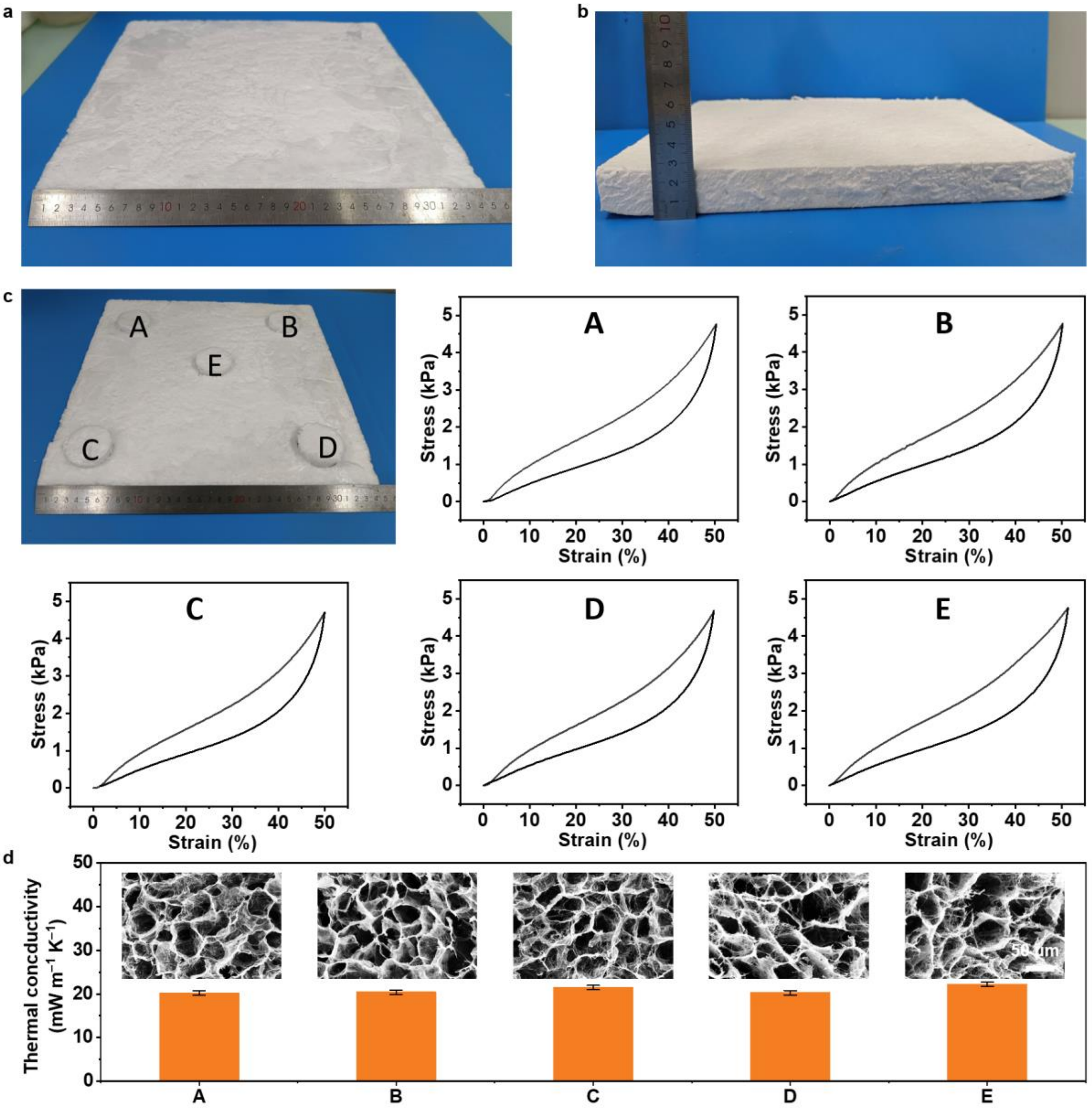

Supplementary Fig. 7. Performance of different parts at a large-scale sample. a In-plane optical images of large-scale samples. **b** Images of ASNF aerogels on thickness direction. **c** Optical images showing the sampling point. **c** Compressive stress-strain curves of ASNF aerogels at different parts from A to E. **d** Micromorphology and thermal conductivity of ASNF aerogels at different parts from A to E.

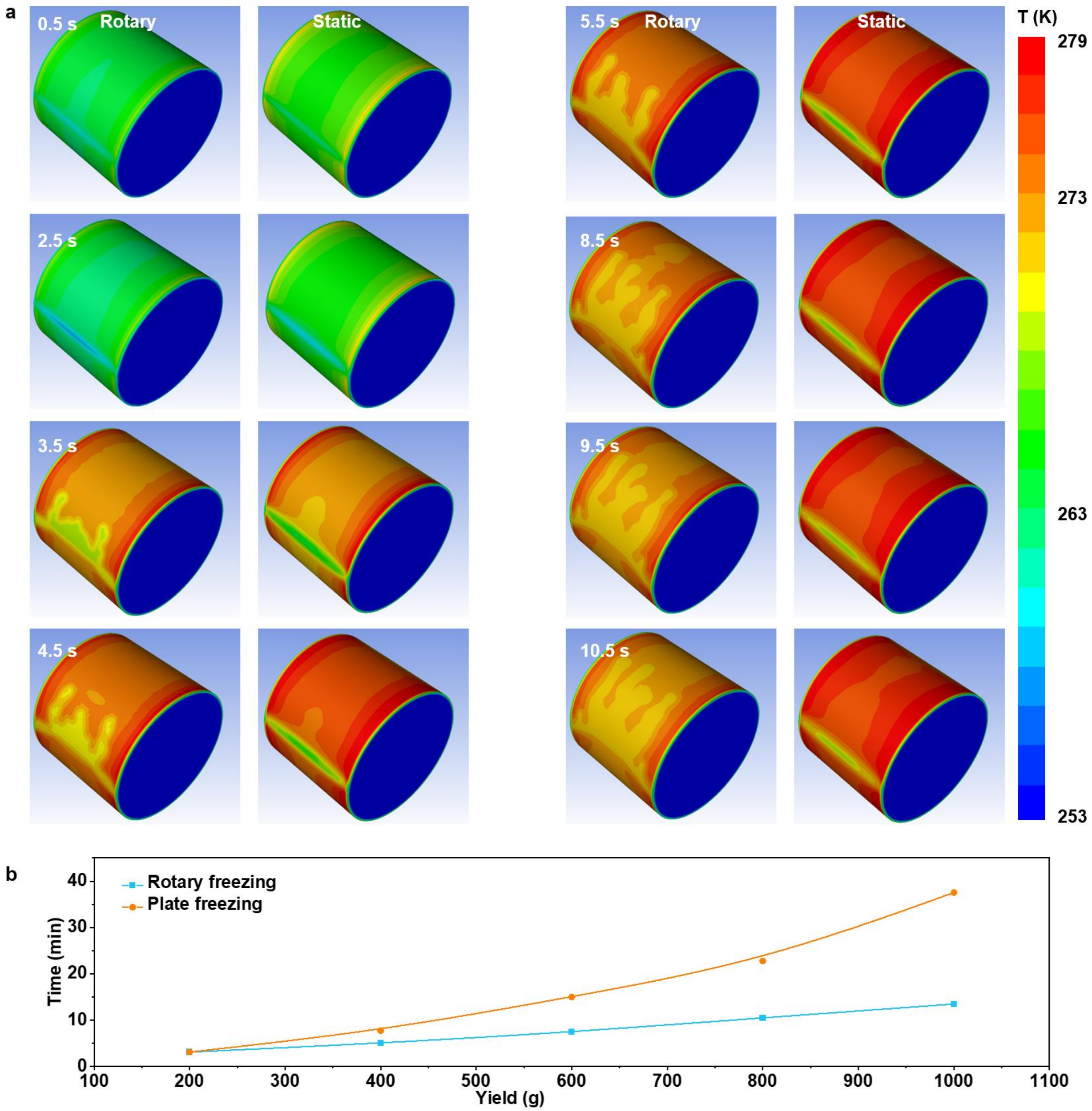

Supplementary Fig. 8. Comparison of heat transfer efficiency between rotary and static states. a CFD simulations on the surface of the rotary or static cryogenic drum. **b** Comparison of ice crystal production rate between the rotary drum and static plate with the same surface area.

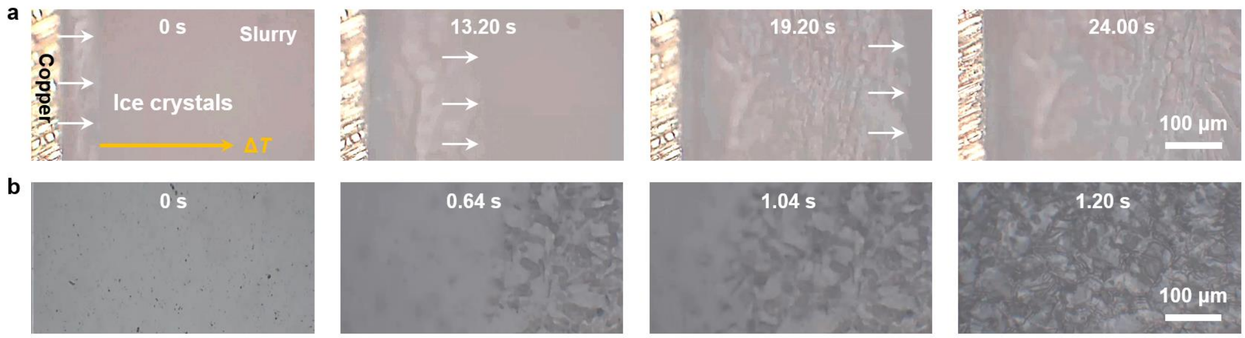

Supplementary Fig. 9. Proposed freezing process. *In situ* observation on directional freezing **(a)** and crushed ice casting **(b)** using an optical microscope.

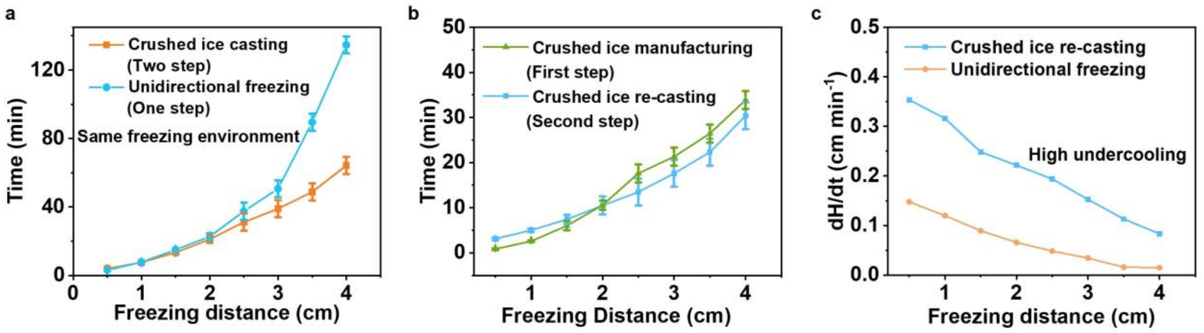

Supplementary Fig. 10. Comparison of time consumption between crushed ice casting and unidirectional freezing methods. a Total production time comparison. **b** Time consumption of crushed ice manufacturing (first step) and crushed ice re-casting steps (second step). **c** Freezing rate of crushed ice re-casting and unidirectional freezing.

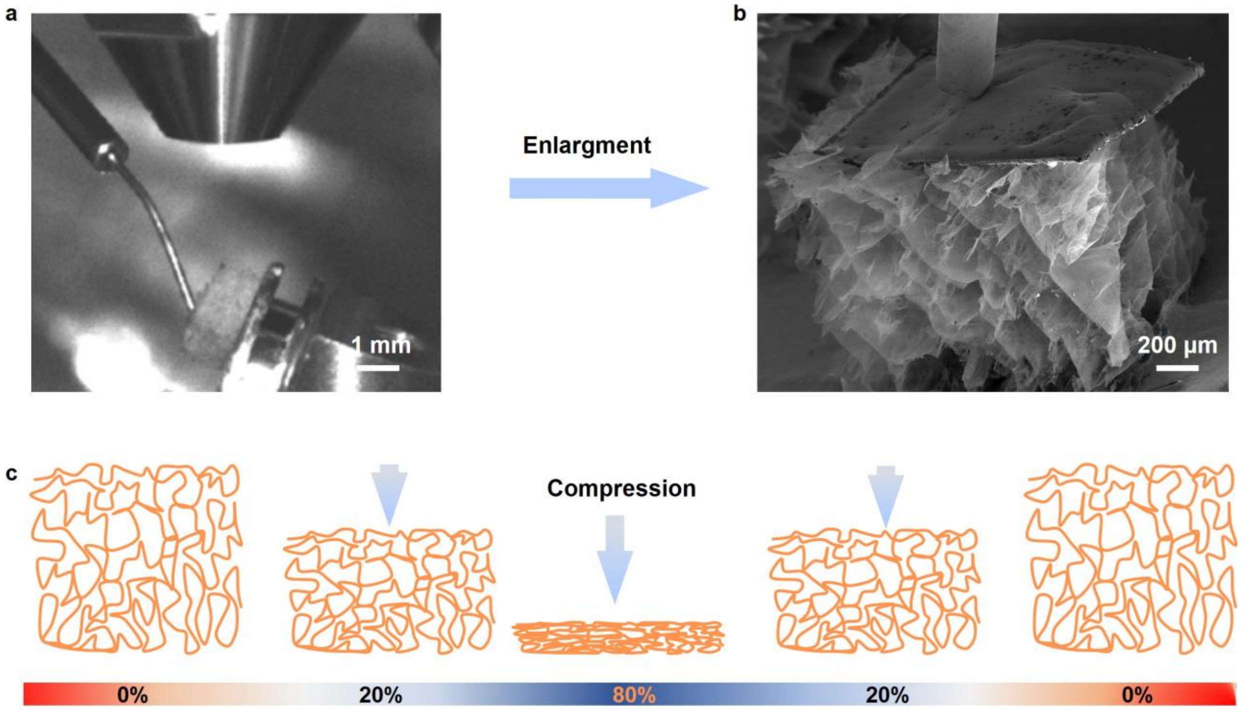

Supplementary Fig. 11. *In-situ* mechanical test. a-b Overlays and enlargement of the *in-situ* SEM images for the full compress-release cycles. From all directions, the morphology of ASNF aerogels is essentially the same, indicating the isotropic structure of the materials. **c** Evolution of cell wall morphology in a complete compression-release cycle.

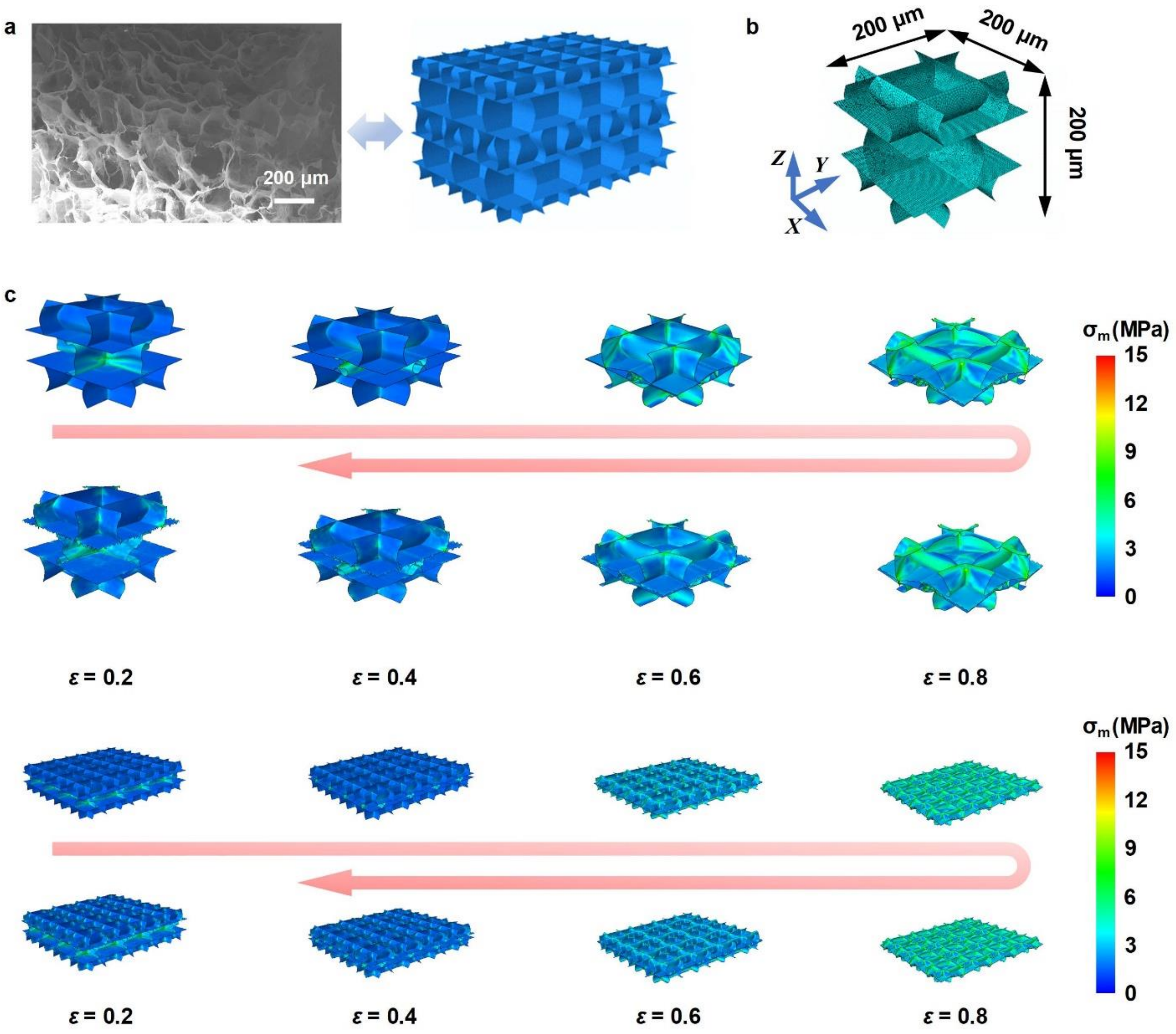

Supplementary Fig. 12. Mechanical simulation. a SEM image shows the separate cellular structures in the tangential cross-section. **b** FEM model created to represent the architectures. **c** Mise stress distributions in the FEA model when compressed to 0.2, 0.4, 0.6 and 0.8.

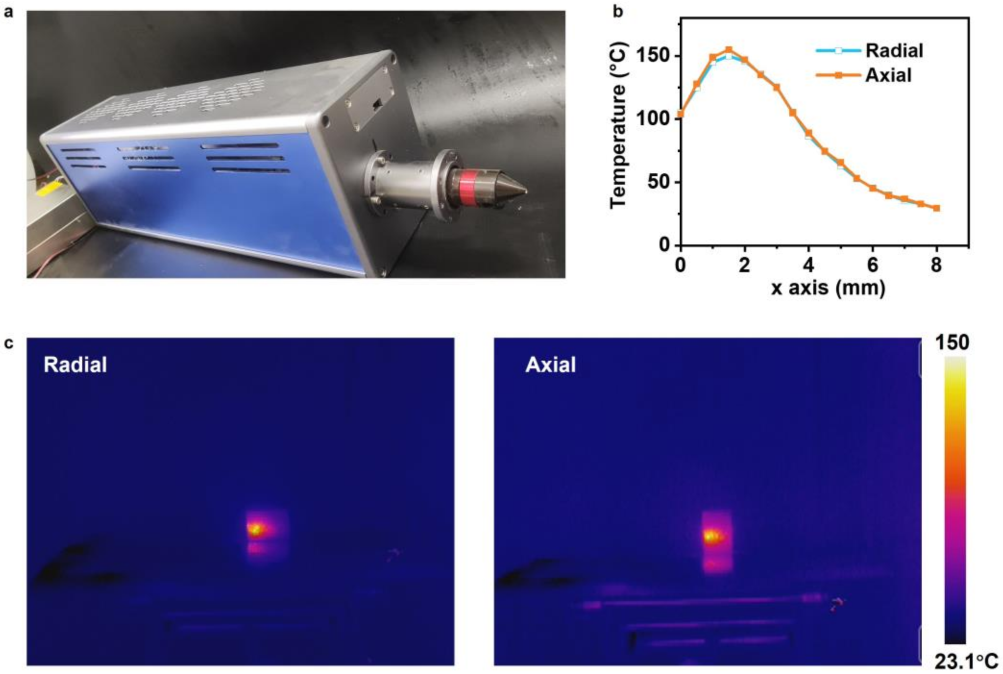

Supplementary Fig. 13. Isotropic characterization. a Collimated 820-nm heat source with a spot size of 3 mm and an input power of 0.35 W. **b** Temperature profile for the samples during radial and axial laser penetration. **c** Infrared images of the samples during radial and axial laser penetration.

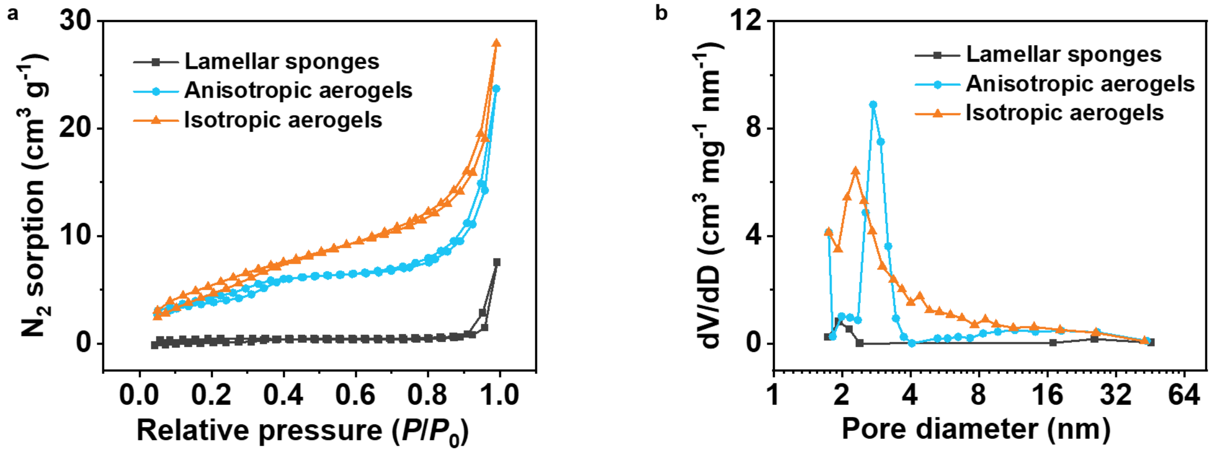

Supplementary Fig. 14. Brunauer-Emmet-Teller analysis. a N_2 sorption isotherms of different materials. **b** Pore size distribution derived from Barrett–Joyner–Halenda analysis.

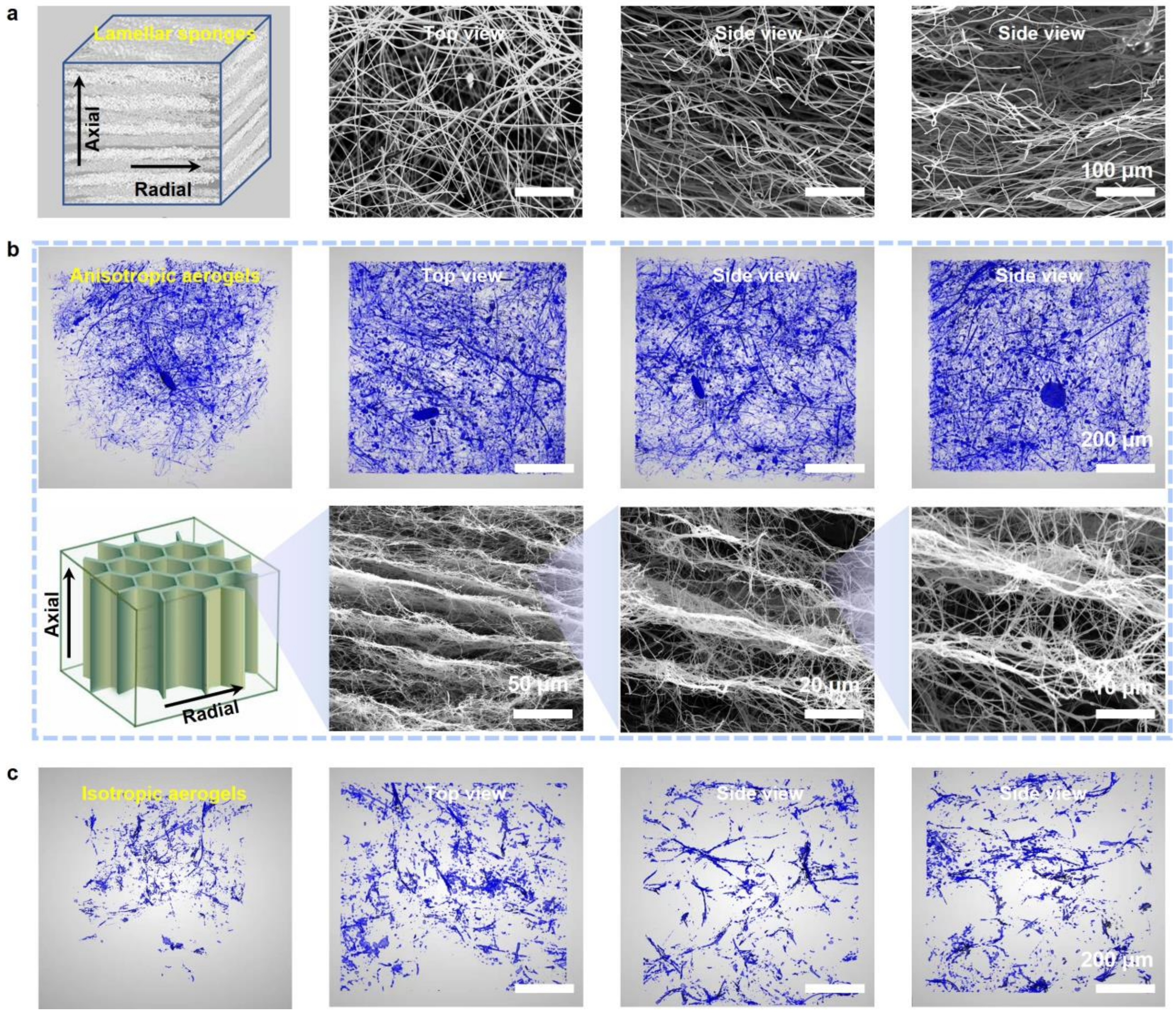

Supplementary Fig. 15. Structure comparison of different porous materials. a SEM images of the ASNF lamellar sponges from the different views. **b** 3D reconstruction of the ASNF anisotropic aerogels from X-ray microtomography (upper). SEM images from the top view at different magnifications (bottom). **c** 3D structure of the ASNF isotropic aerogels from X-ray microtomography.

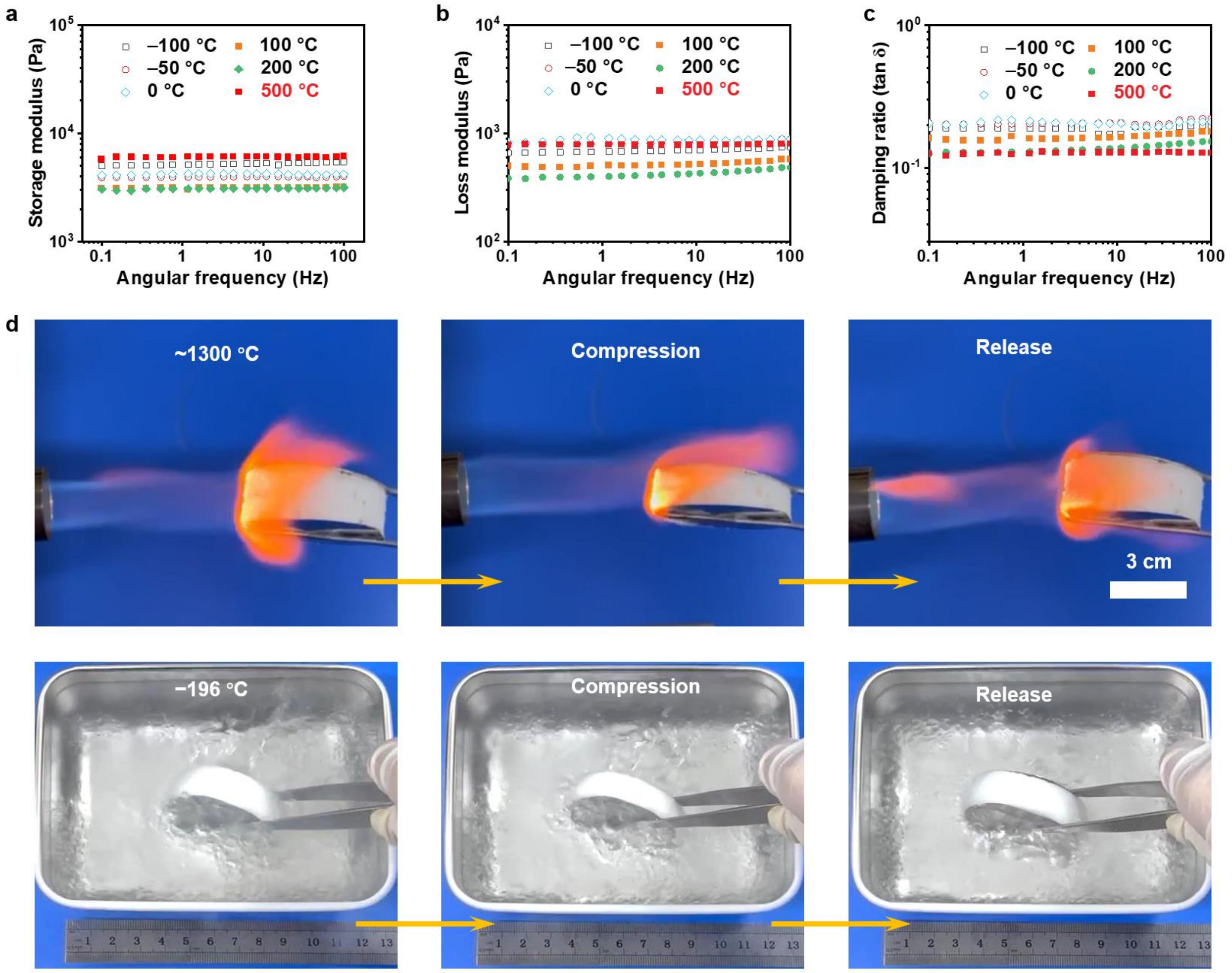

Supplementary Fig. 16. Temperature-invariant mechanical performances. a-c Storage modulus, loss modulus, and damping ratio as a function of angular frequency in the temperature range of −100 to 500 °C. **d** Compression and release processes of ASNF aerogels when exposed to butane blowtorch flame and liquid nitrogen.

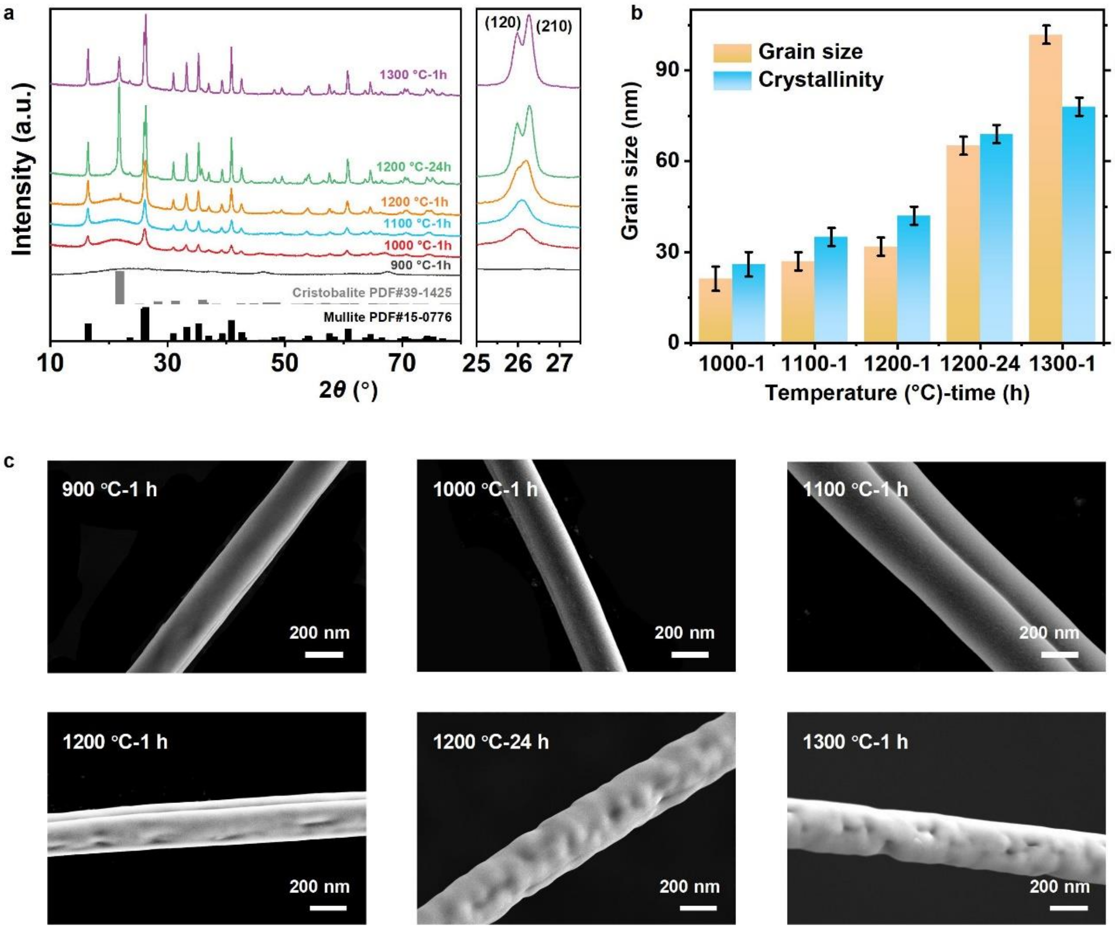

Supplementary Fig. 17. Phase changes at different temperatures. a XRD results of ASNF aerogels after annealing at different temperatures. **b** Corresponding grain size and crystallinity obtained from XRD results after calcination at 900, 1000, 1100, 1200, and 1300 °C for different time. **c** SEM images after calcination at 1000, 1100, 1200, and 1300 for different time.

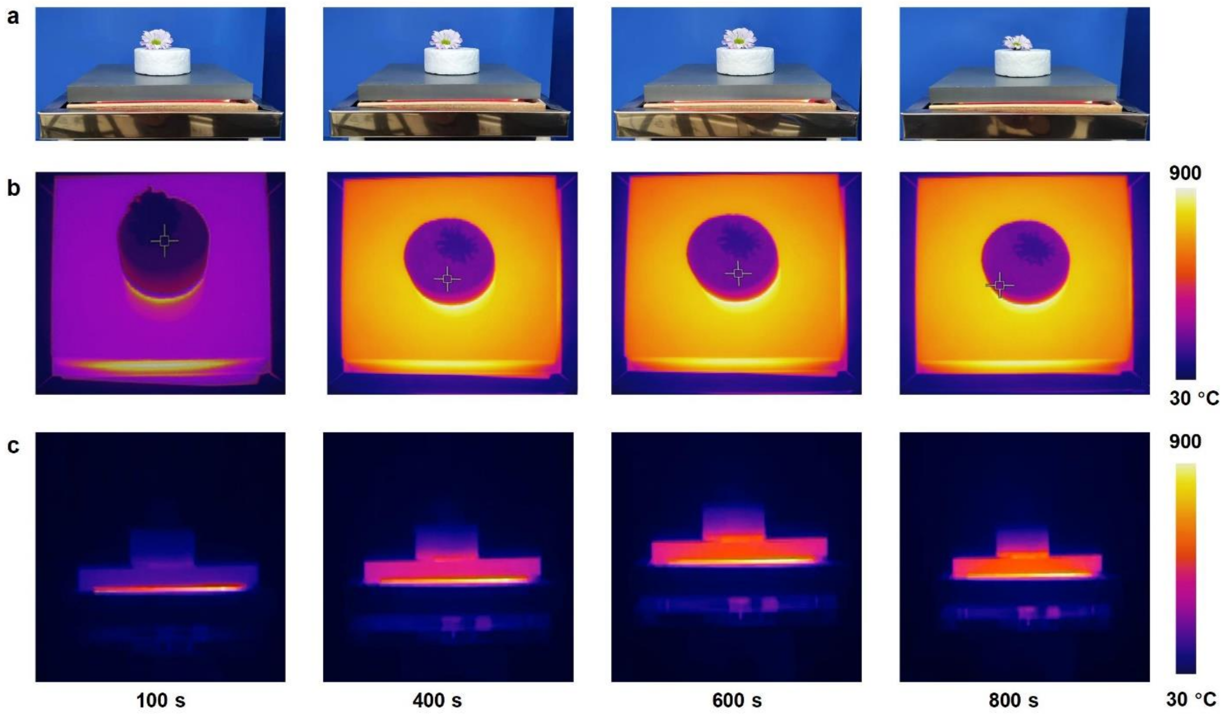

Supplementary Fig. 18. Time-dependent optical (a) and infrared images (b and c) of a 3-cm-thick aerogel placed on a hot plate at 900 °C, which is made of 2 cm thick silicon carbide plate placed on the resistor with a heating power of 2 kW.

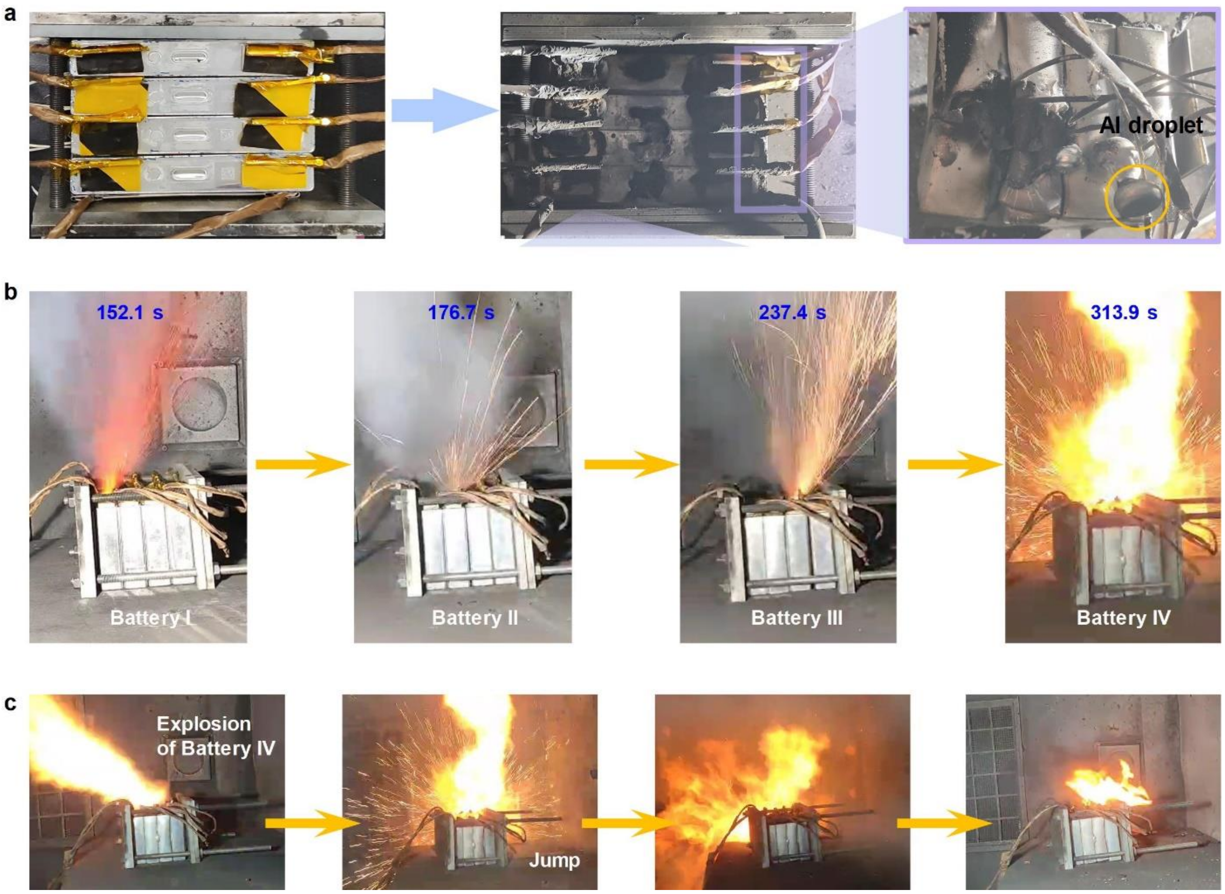

Supplementary Fig. 19. Overheating-induced thermal propagation tests in an unprotected four–cell battery module. a Battery module structure before and after TR. **b** TR time of each battery. **c** Explosion process of battery IV.

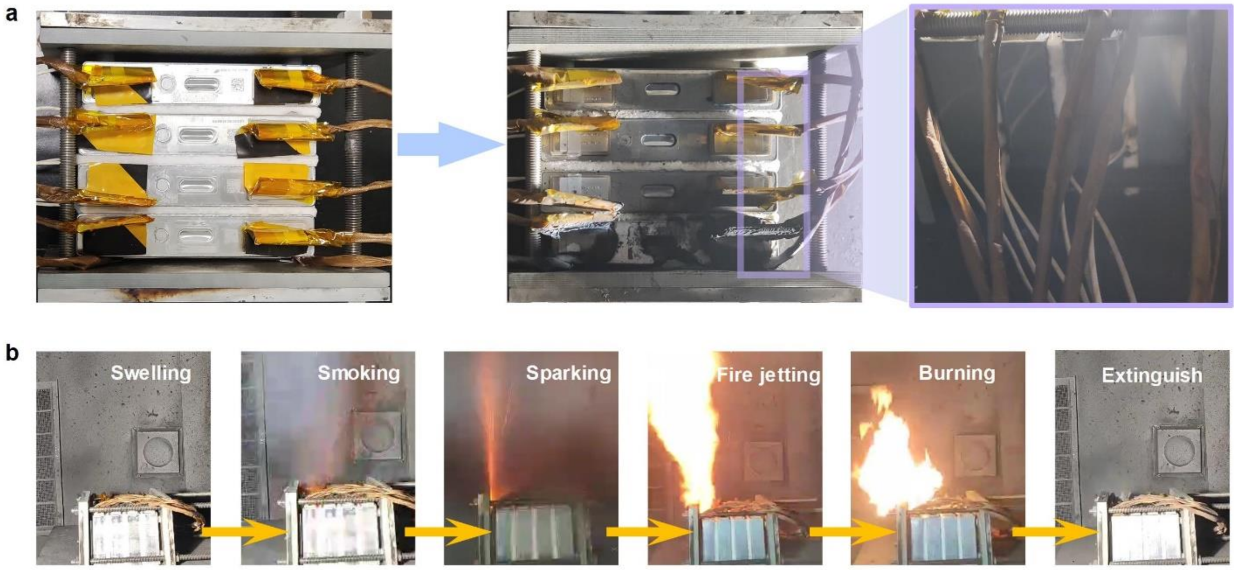

Supplementary Fig. 20. Overheating-induced thermal propagation tests in a four–cell battery module protected by ASNF aerogels. a Battery module structure before and after TR test. **b** TR process of battery I.

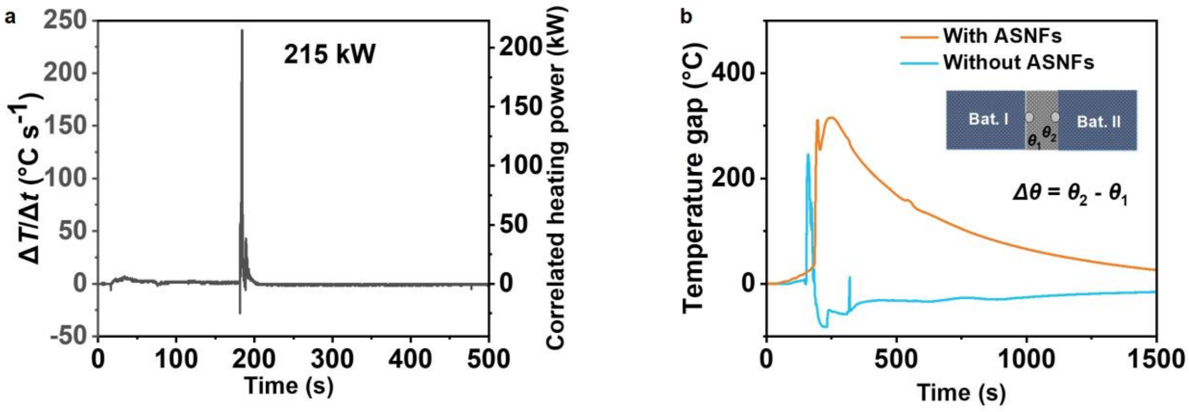

Supplementary Fig. 21. Analysis of TR propagation processes protected by ASNF aerogels. **a** Differential result of the temperature response profile for the back surface of Battery I and the correlated heating power. **b** Temperature gap between Battery I and II *versus* time.

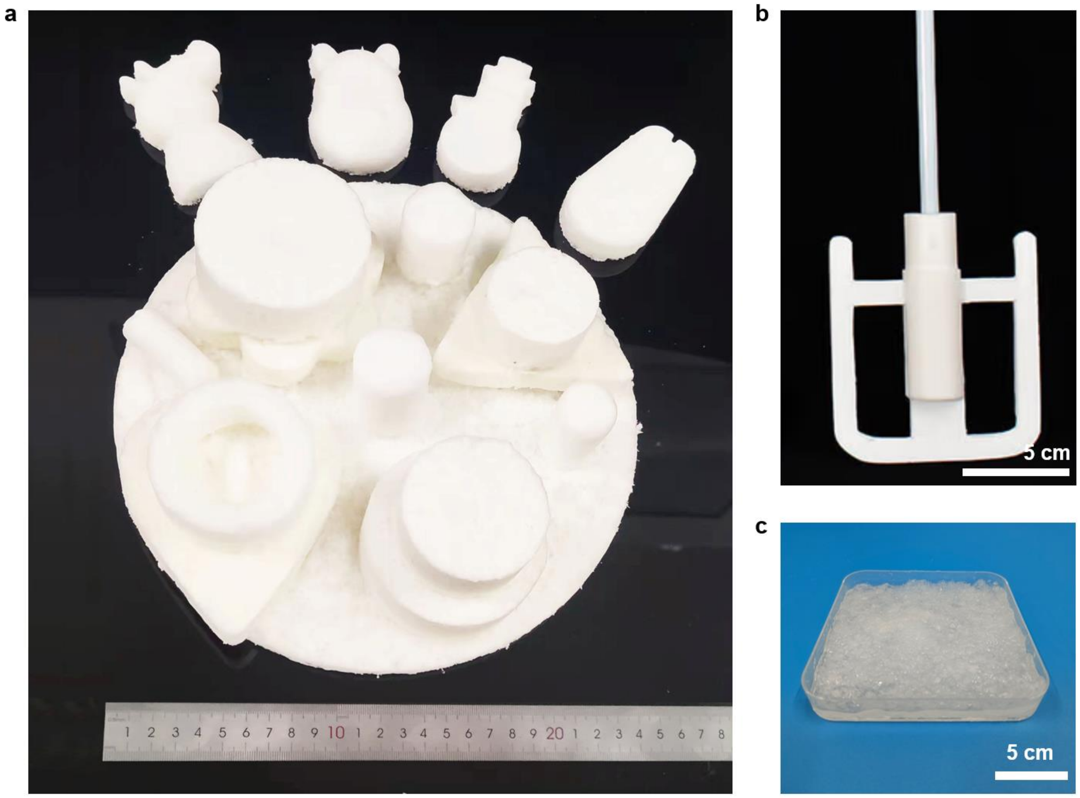

Supplementary Fig. 22. Optical images of samples and agitator paddles. a ASNF aerogels with diverse shapes. The ASNF aerogels with an isotropic structure could be molded into arbitrary shapes to fit the needs of the current diversified battery or module forms. **b** Customized agitator paddles for mixing crushed ice and nanofiber dispersion. **c** Optical images of crushed-ice-dispersion mixture.

Supplementary Table 1. Thermal conductivity and elastic modulus of the nanofiber aerogels with different bulk density.

Density (mg cm^{-3})	Thermal conductivity (mW m^{-1} K^{-1})	Young's modulus (kPa)
0.6	24.01	0.25
1.0	24.38	0.50
2.5	25.00	0.98
5.0	26.00	3.95
12.0	28.28	8.17
16.0	29.21	10.36

Supplementary Table 2. Whole process time from raw materials to products comparison between isotropic ASNF aerogels and the traditional fiber felt/silica aerogel composites (Use 300 × 300× 10 mm^3 sample as standard).

Isotropic ASNF aerogels*		Fiber felt/silica aerogel composites**	
Procedures	Time (h)	Procedures	Time (h)
Nanofiber preparation	~24.0	Gel preparation	~24.5
Dispersion preparation	~0.1	Aging	~12.0
Crushed ice casting (Two steps)	~0.5	Modification-replacement	~36.0
Freeze drying	~48.0	Drying	~24.0 (Estimate)
Total	~72.6	Total	~96.5

*We have ignored the preparation time of excipients.
** We have ignored the preparation time of fiber felts.

Supplementary Table 3. Pore structural parameters of the samples.

Samples	BET Specific surface area (m^2 g^{-1})	BJH Pore volume (cm^3 g^{-1})	Average pore diameter (nm)
Lamellar sponges	1.54	0.0116	2.19
Anisotropic aerogels	16.15	0.0359	3.28
Isotropic aerogels	21.89	0.0444	3.86

Supplementary Table 4. Comparison of materials prepared in this study with similar thermally insulating structures.

**Materials**	**Temperature**	**Thermal Conductivity ($mW\ m^{-1}\ K^{-1}$)**	**Ref.**
hBN aerogels	900	24.0	Ref. [10]
Ceramic microfiber sponges	1200	34.0	Ref. [11]
SiO_2 aerogels	400	15.9	Ref. [12]
SiO_2 nanofiber aerogels	1100	25.0	Ref. [13]
SiC@SiO_2 nanowire aerogels	1000	14.0	Ref. [14]
Carbon nanotube Aerogels	500	23.0	Ref. [8]
Graphene/Al_2O_3	500	30.0	Ref. [15]
Nanowood	280	30.0	Ref. [16]
Nanocellulose/graphene oxide	280	15.0	Ref.[17]
**ASNF aerogels**	**1200**	**20.0**	**Our work**

Supplementary Table 5. The parameters of drum and liquid film.

Item	Value
Rotation axis direction /(x, y, z)	(1,0,0)
Rotating speed of drum /rad·min^{-1}	50
Initial drum temperature /K	253.15
Initial liquid film temperature /K	293.15
Liquid film thickness/mm	1-3

Supplementary Movie 1.

Rapid freezing nanofiber dispersion on the surface of the rotating cryogenic drum and scraping them into crushed ice.

Supplementary Movie 2.

Compression–recovery testing at room temperature.

Supplementary Movie 3.

Computational fluid dynamics simulations on the surface of the rotary or static cryogenic drum.

Supplementary Movie 4.

The formation mechanism for different aerogels from directional freeze casting or crushed ice casting methods.

Supplementary Movie 5.

In-situ observation of ice crystal growth on a copper surface or in a crushed-ice-slurry system.

Supplementary Movie 6.

In-situ mechanical compression testing and simulation *via* a nonlinear finite element model.

Supplementary Movie 7.

3D reconstruction of the isotropic and anisotropic ASNF aerogels from X-ray microtomography.

Supplementary Movie 8.

Compression–recovery testing under extreme conditions.

Supplementary Movie 9.

Time-dependent optical and infrared images of a 3−cm−thick ASNF aerogel placed on a hot plate at 900 ℃.

Supplementary Movie 10.

Domino-effect cell-to-cell deflagration-propagation process in a practical lithium-ion battery module consisting of NCM811 cathode.

Supplementary Movie 11.

Practical domino-risk-free lithium-ion battery modules consisting of NCM811 cathode enabled by ASNF aerogels.

References

1. Li L*, et al.* Nanograin–glass dual-phasic, elasto-flexible, fatigue-tolerant, and heat-insulating ceramic sponges at large scales. *Mater Today* **54**, 72-82 (2022).
2. Xie C, He L, Shi Y, Guo Z-X, Qiu T, Tuo X. From Monomers to a Lasagna-like Aerogel Monolith: An Assembling Strategy for Aramid Nanofibers. *ACS Nano* **13**, 7811-7824 (2019).
3. Saito T, Kimura S, Nishiyama Y, Isogai A. Cellulose nanofibers prepared by TEMPO-mediated oxidation of native cellulose. *Biomacromolecules* **8**, 2485-2491 (2007).
4. Lin S*, et al.* Room-temperature production of silver-nanofiber film for large-area, transparent and flexible surface electromagnetic interference shielding. *npj Flexible Electronics* **3**, (2019).
5. Wang J, Liu W, Song X, Ma Y, Huang Y. Effects of added polyvinyl pyrrolidone on morphology and microstructure of multiple-phase mullite nanofibers. *Ceram Int* **44**, 15418-15427 (2018).
6. He YL, Xie T. Advances of thermal conductivity models of nanoscale silica aerogel insulation material. *Appl Therm Eng* **81**, 28-50 (2015).
7. Carson JK, Lovatt SJ, Tanner DJ, Cleland AC. Thermal conductivity bounds for isotropic, porous materials. *Int J Heat Mass Transfer* **48**, 2150-2158 (2005).
8. Zhan HJ*, et al.* Biomimetic carbon tube aerogel enables super-elasticity and thermal insulation. *Chem* **5**, 1871-1882 (2019).
9. Zeng SQ, Hunt A, Greif R. Transport properties of gas in silica aerogel. *J Non-Cryst Solids* **186**, 264-270 (1995).
10. Xu X*, et al.* Double-negative-index ceramic aerogels for thermal superinsulation. *Science*

363, 723-727 (2019).

11. Jia C*, et al.* Highly compressible and anisotropic lamellar ceramic sponges with superior thermal insulation and acoustic absorption performances. *Nat Commun* **11**, 3732 (2020).

12. Zhao S*, et al.* Additive manufacturing of silica aerogels. *Nature* **584**, 387-392 (2020).

13. Si Y, Wang X, Dou L, Yu J, Ding B. Ultralight and fire-resistant ceramic nanofibrous aerogels with temperature-invariant superelasticity. *Sci Adv* **4**, eaas8925 (2018).

14. Su L*, et al.* Anisotropic and hierarchical SiC@SiO_2 nanowire aerogel with exceptional stiffness and stability for thermal superinsulation. *Sci Adv* **6**, eaay6689 (2020).

15. Zhang Q*, et al.* Flyweight, superelastic, electrically conductive, and flame-retardant 3D multi-nanolayer graphene/ceramic metamaterial. *Adv Mater* **29**, 1605506 (2017).

16. Li T*, et al.* Anisotropic, lightweight, strong, and super thermally insulating nanowood with naturally aligned nanocellulose. *Sci Adv* **4**, eaar3724 (2018).

17. Wicklein B*, et al.* Thermally insulating and fire-retardant lightweight anisotropic foams based on nanocellulose and graphene oxide. *Nat Nanotechnol* **10**, 277-283 (2015).

18. Li L*, et al.* Thermal-switchable, trifunctional ceramic-hydrogel nanocomposites enable full-lifecycle security in practical battery systems. *ACS Nano* **16**, 10729-10741 (2022).

19. Li L*, et al.* Thermal-responsive, super-strong, ultrathin firewalls for quenching thermal runaway in high-energy battery modules. *Energy Storage Mater* **40**, 329-336 (2021).
