# Supplementary material for: Large-scale assembly of isotropic nanofiber aerogels based on columnar-equiaxed crystal transition
Source: Nat Commun. 2023 Sep 5;14:5410. doi: 10.1038/s41467-023-41087-y (PMC10480443; doi:10.1038/s41467-023-41087-y)
Supplement: Supplementary file 3 — Description of Additional Supplementary Files [file 41467_2023_41087_MOESM3_ESM.pdf]

Description of Additional Supplementary Files

File Name: Supplementary Movie 1
Description: Rapid freezing nanofiber dispersion on the surface of the rotating cryogenic drum and scraping them into crushed ice.

File Name: Supplementary Movie 2
Description: Compression–recovery testing at room temperature.

File Name: Supplementary Movie 3
Description: Computational fluid dynamics simulations on the surface of the rotary or static cryogenic drum.

File Name: Supplementary Movie 4
Description: The formation mechanism for different aerogels from directional freeze casting or crushed ice casting methods.

File Name: Supplementary Movie 5
Description: *In-situ* observation of ice crystal growth on a copper surface or in a crushed-ice-slurry system.

File Name: Supplementary Movie 6
Description: *In-situ* mechanical compression testing and simulation *via* a nonlinear finite element model.

File Name: Supplementary Movie 7
Description: 3D reconstruction of the isotropic and anisotropic ASNF aerogels from X-ray microtomography.

File Name: Supplementary Movie 8
Description: Compression–recovery testing under extreme conditions.

File Name: Supplementary Movie 9
Description: Time-dependent optical and infrared images of a 3–cm–thick ASNF aerogel placed on a hot plate at 900 °C.

File Name: Supplementary Movie 10
Description: Domino-effect cell-to-cell deflagration-propagation process in a practical lithium-ion battery module consisting of NCM811 cathode.

File Name: Supplementary Movie 11
Description: Practical domino-risk-free lithium-ion battery modules consisting of NCM811 cathode enabled by ASNF aerogels.
